# Effects of short-term hypercaloric nutrition on orthostatic tolerance in healthy individuals: a randomized controlled crossover study

**DOI:** 10.1007/s10286-022-00900-2

**Published:** 2022-10-05

**Authors:** Riccardo De Gioannis, Ann C. Ewald, Darius A. Gerlach, Karsten Heusser, Fabian Hoffmann, Petra Frings-Meuthen, Martina Heer, Jens Tank, Jens Jordan

**Affiliations:** 1 Institute of Aerospace Medicine, German Aerospace Center (DLR), Linder Hoehe, 51147 Cologne, Germany; 2 Faculty of Medicine, Department III for Internal Medicine, Heart Center, University Hospital of Cologne, Cologne, Germany; 3 IU International University of Applied Sciences, Erfurt, Germany; 4 Chair of Aerospace Medicine, University of Cologne, Cologne, Germany

**Keywords:** Diet, Metabolism, Hypercaloric, Neurally mediated syncope, Sympathetic nervous system

## Abstract

Reduced-caloric intake lowers blood pressure through sympathetic inhibition, and worsens orthostatic tolerance within days. Conversely, hypercaloric nutrition augments sympathetic activity and blood pressure. Because dietary interventions could be applied in patients with syncope, we tested the hypothesis that short-term hypercaloric dieting improves orthostatic tolerance. In a randomized crossover trial, 20 healthy individuals (7 women, 26.7 ± 8 years, 22.6 ± 2 kg/m^2^) followed a 4-day hypercaloric (25% increase of energy intake by fat) or normocaloric nutritional plan, with a washout period of at least 23 days between interventions. We then performed head-up tilt table testing with incremental lower body negative pressure while recording beat-by-beat blood pressure and heart rate. The primary endpoint was orthostatic tolerance defined as time to presyncope. Time to presyncope during combined head-up tilt and lower body negative pressure did not differ between hypercaloric and normocaloric dieting (median 23.19 versus 23.04 min, ratio of median 1.01, 95% CI of ratio 0.5–1.9). Heart rate, blood pressure, heart rate variability, and blood pressure variability in the supine position and during orthostatic testing did not differ between interventions. We conclude that 4 days of moderate hypercaloric nutrition does not significantly improve orthostatic tolerance in healthy individuals. Nevertheless, given the important interaction between energy balance and cardiovascular autonomic control in the brain, caloric intake deserves more attention as a potential contributor and treatment target for orthostatic intolerance.

## Introduction

Neurally mediated syncope is a common reason for emergency room admissions [28], but can also occur in astronauts returning from space [19]. On Earth, the condition is relatively benign except for fall-related injuries, yet affected patients may experience a strong negative impact on quality of life [21]. However, neurally mediated syncope could have catastrophic consequences when setting foot on another celestial body. Nonpharmacological interventions such as increasing fluid intake or counterpressure maneuvers are commonly recommended but may not suffice [6]. Pharmacological therapies are of limited efficacy [22], and few patients with neurally mediated syncope are eligible for cardiac pacemaker implantation [8]. Changes in the balance between parasympathetic and sympathetic activity towards parasympathetic predominance seem to set off syncope. Increased caloric intake activates sympathetic preganglionic neurons while inhibiting parasympathetic efferents [34]. In obesity, chronic caloric surplus raises sympathetic activity and blood pressure [14] while improving orthostatic tolerance [24]. Chronic caloric deficiency elicits the opposite response in individuals with low body mass index and in patients with anorexia nervosa [17, 23]. A few days of caloric restriction are sufficient in reducing sympathetic outflow and orthostatic tolerance in both obese and lean individuals [2, 12]. Given the intense cross talk between caloric intake and autonomic control of orthostatic tolerance, we hypothesized that increased caloric intake over 4 days, which could be reasonably implemented in space and in patients on Earth, improves orthostatic tolerance in healthy people. Orthostatic tolerance testing through head-up tilt testing with incremental lower body negative pressure (LBNP) [10] in healthy people has proven useful when screening for potential neurally mediated syncope treatments [7, 29, 30].

## Methods

### Subjects

Healthy people aged 18–40 years, with a body mass index between 18 and 25 kg/m^2^ and a resting heart rate > 55 beats per minute (bpm) were eligible for this study. Main exclusion criteria were recent body mass changes > 3 kg, history of syncope or cardiac arrhythmia, smoking, and alcohol or drug abuse. We ruled out preexisting diseases by obtaining a detailed history, and performing a physical examination, a 12-lead electrocardiogram, blood pressure recordings, and taking blood samples for routine laboratory tests. Subjects on medication that might impact study outcomes were excluded. We obtained written informed consent before inclusion in the study. The study was approved by the ethics committee of the Medical Board North Rhine (Nr. 2018071) and was performed in accordance with the declaration of Helsinki (2013). We prospectively registered the study on DRKS (German Clinical Trials Register) (registration number: DRKS00020750).

### Study design and protocol

**Fig. 1:**
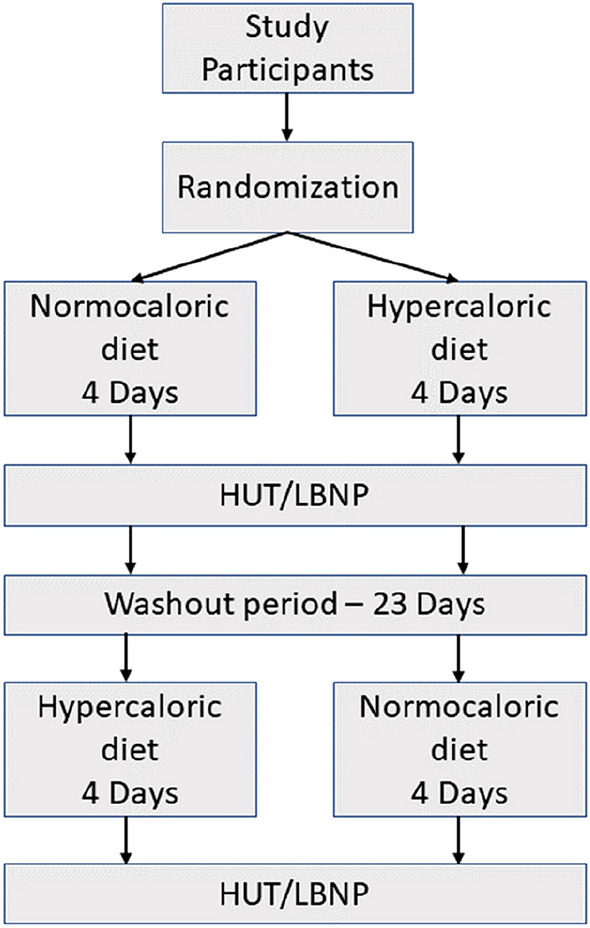
Study protocol

This randomized cross-over study was conducted at the Institute of Aerospace Medicine of the German Aerospace Center in Cologne, Germany. Participants followed hypercaloric or normocaloric nutritional plans over 4 days, with a washout period of at least 23 days between interventions (Fig. 1). In the first 3 days of each dietary intervention, we provided all meals; however, participants stayed at home. In the subsequent 2 days, participants were admitted to the clinical research ward at the :envihab facility and received all meals under highly controlled conditions. At the end of each dietary intervention, we assessed orthostatic tolerance through a combination of head-up tilt testing and incremental lower body negative pressure, as described previously [10, 30]. The randomization was conducted by assigning a random number to each subject generated in Excel.

### Dietary intervention

We determined individual total energy expenditure based on individually measured resting metabolic rate and estimated physical activity level. We assessed resting metabolic rate and substrate oxidation through indirect calorimetry (Quark RMR, COSMED). We determined individual energy requirements over 24 h via the Freiburger questionnaire. Caloric content of the normocaloric diet corresponded to individual total energy expenditure. Caloric intake was increased by 25% above total energy expenditure during the hypercaloric diet. We increased energy content by raising fat intake, while carbohydrate, fiber, and protein were kept constant. Fluids, as well as dietary sodium and potassium intake, were kept constant during both dietary phases. Both dietary plans included commercially available food. To ensure compliance to the dietary intervention during the outpatient phases, we provided all food items as well as nutritional protocols. Food had to be weighed by study participants to the nearest gram and noted in the protocol. These logs were then analyzed (PRODI, Nutri-Science GmbH) to calculate nutrient intake (Table 2). Participants and investigators were blinded for the dietary intervention; only the project lead and the metabolic kitchen knew the respective diets.

### Orthostatic tolerance testing

Testing was conducted in the morning hours after an overnight fast. We collected blood samples 30 min before orthostatic testing through an indwelling venous catheter inserted the previous day. We measured continuous finger blood pressure, brachial blood pressure, and electrocardiogram. Subjects rested in supine position for 15 min and were then tilted head up to a 60° position for 20 min. Subsequently, we applied incremental lower body negative pressure steps (−20, −40, and −60 mmHg), each lasting 10 min. The test was terminated when brachial systolic blood pressure decreased below 80 mmHg (or < 90 mmHg and rapidly decreasing), when finger blood pressure and heart rate decreased simultaneously, when participants reported presyncopal symptoms, when syncope occurred, or when subjects requested to abort the test. We defined orthostatic tolerance as time to presyncope expressed in minutes [10]. Data acquisition and analysis were conducted as described elsewhere [31].

### Predefined endpoints

The primary endpoint of the study was the change in time to presyncope. Exploratory endpoints included blood pressure, heart rate, heart rate variability, and baroreflex sensitivity before and during orthostatic testing.

### Sample size justification

In a previous study in a similar sample, the improvement in time to presyncope had a standard deviation of 4.8 min [30]. In the present cross-over study, we expected to find a 2-min improvement in time to presyncope. Using the tool GPower 3.1, we calculated that 20 participants would have to complete the study to reject the null hypothesis with a type I error probability of 0.05 and a type II error probability of 0.80.

### Statistics

According to a prospective data analysis plan, we used a general linear model to assess the effect of the hypercaloric intervention and linear regression analyses to detect correlation among variables. Within-individual differences were tested with a paired *t*-test. Differences were considered significant with *p* < 0.05. Data are reported as mean ± SEM.

## Results

**Fig. 2:**
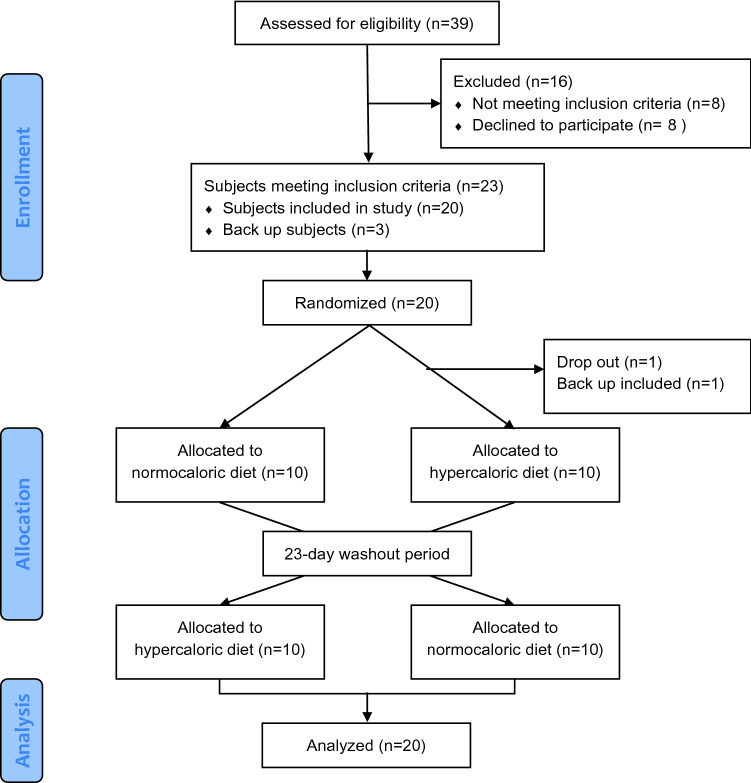
Consort sheet to illustrate the recruitment process

We screened 39 people (Fig. 2). Of those, 23 fulfilled all inclusion and exclusion criteria and entered the study. Twenty study participants completed all study visits and were included in the analysis (Table 1). No serious adverse events occurred.

**Table 1:** Study population

Subject	Sex	Age (years)	Height (cm)	BMI Normocaloric (kg/m^2^)	Weight Normocaloric (kg)	BMI Hypercaloric (kg/m^2^)	Weight Hypercaloric (kg)
A	m	27	179.5	24.6	79.3	24.8	79.8
B	w	19	167.0	21.0	58.5	21.2	59.0
C	m	27	188.0	23.3	82.2	23.2	81.9
D	m	23	172.0	22.8	67.4	22.8	67.5
E	m	30	183.0	23.9	79.9	24.2	80.9
F	w	32	159.5	24.6	62.6	24.9	63.2
G	w	23	172.5	19.1	56.8	19.2	57.0
H	m	34	181.0	22.0	72.1	22.0	72.2
I	m	23	185.0	25.4	87.0	25.6	87.5
K	m	25	172.0	21.7	64.1	21.8	64.4
L	m	36	176.0	25.2	77.9	25.2	78.1
M	m	23	182.0	24.3	80.4	23.6	78.1
N	w	22	162.5	19.4	51.3	19.6	51.8
O	w	22	173.5	19.8	59.6	19.9	59.9
P	m	33	175.5	22.6	69.5	22.7	69.9
R	w	29	173.0	20.4	61.1	20.5	61.2
S	m	21	175.5	23.9	73.6	23.9	73.7
T	m	25	186.5	26.0	90.4	25.6	88.9
U	w	36	163.0	20.6	54.7	20.5	54.5
W	m	24	180.5	22.2	72.2	22.5	73.2

*m* man, *w* woman

Dietary energy content and composition during normocaloric and hypercaloric dieting are presented in Table 2. As planned, the only difference between dietary interventions was an increase in dietary fat during the hypercaloric phase. Sodium content of the diet was 1.898 ± 0.128 and 1.906 ± 0.132 mmol per kg of body mass per day in the normocaloric and hypercaloric phase, respectively (*p* = 0.85). Fluid intake was adjusted to the individual needs of each participant and kept constant during the two phases.

**Table 2:** Diet composition

Characteristic	Hypercaloric *N* = 20	Normocaloric *N* = 20	*p*-Value
Calories (cal)	2922 (531)	2372 (439)	0.001
Protein (g)	85 (13)	84 (13)	0.9
Fat (g)	132 (23)	74 (13)	< 0.001
Carbohydrates (g)	312 (65)	310 (65)	> 0.9
Fiber (g)	35 (5)	35 (5)	> 0.9

Values are expressed as mean (standard deviation)

**Fig. 3:**
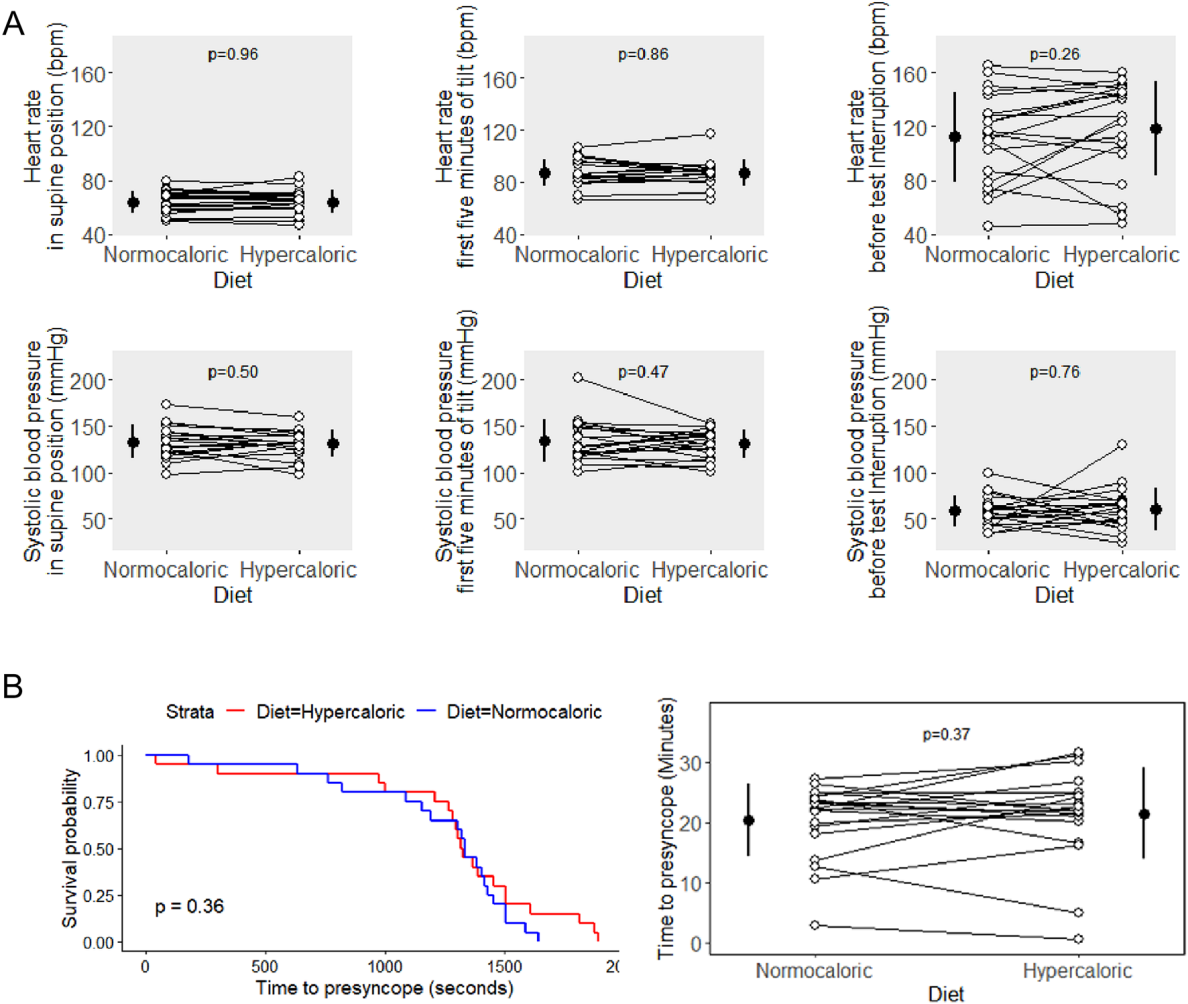
**A** Heart rate and systolic blood pressure at baseline, 5 min after tilt and before test interruption. **B** Time to presyncope

Figure 3 (left panels) shows individual supine heart rate and blood pressure data. Supine blood pressure was 133 ± 17.6/66.4 ± 10.4 mmHg on the normocaloric and 131 ± 13.9/64.2 ± 8.7 mmHg on the hypercaloric diet (systolic *p* = 0.50, diastolic *p* = 0.28). Supine heart rate was 64.0 ± 8.2 bpm on the normocaloric and 64.1 ± 8.6 bpm on the hypercaloric diet (*p* = 0.96). Moreover, the hemodynamic response during the initial phase of orthostatic testing did not differ between interventions (Fig. 3—central panels). On average, systolic blood pressure decreased slightly during this phase, while heart rate increased modestly. Diastolic blood pressure was well maintained in both groups.

Time to presyncope during combined head-up tilt and lower body negative pressure, the primary endpoint of the study, did not differ between hypercaloric and normocaloric diets (median 23.3 versus 23.1 min, ratio of median 1.01, 95% CI of ratio 0.5–1.8. Systolic finger blood pressure during the last phase of orthostatic testing did not differ between dietary interventions (normocaloric: 58.6 ± 16.2/32.9 ± 6.7 mmHg; hypercaloric: 60.6 ± 23.1/34.5 ± 11.9 mmHg, not significant for either systolic or diastolic values). The same is true for heart rate before test interruption, which was 112.0 ± 33.1 bpm on the normocaloric and 118 ± 35.0 bpm on the hypercaloric diet (*p* = 0.26).

Dietary caloric intake did not affect heart rate variability, blood pressure variability, or baroreflex sensitivity in the supine or in the upright position (Table 3).

**Table 3:** Heart rate variability, baroreflex sensitivity, and blood pressure variability in supine position and during the first 5 min of tilt

Characteristic	Supine position			First 5 min of tilt		
	Hypercaloric *N* = 20	Normocaloric *N* = 20	*p*-Value	Hypercaloric *N* = 20	Normocaloric *N* = 20	*p* value
LF-HR variability (nu)	66.60 (13.33)	67.95 (14.64)	0.53	84.15 (12.40)	82.80 (12.29)	0.39
HF-HR variability (nu)	33.40 (13.33)	32.05 (14.64)	0.53	15.85 (12.40)	17.20 (12.29)	0.39
Baroreflex sensitivity (ms/mmHg)	15.15 (5.23)	15.30 (6.07)	0.94	6.10 (1.61)	5.75 (1.67)	0.46
LF-systolic blood pressure (mmHg^2^)	6.35 (4.43)	7.25 (4.90)	0.54	26.60 (17.57)	30.55 (18.10)	0.30

## Discussion

The important finding of our study is that a moderate increase in caloric intake for 4 days compared with normocaloric nutrition does not significantly improve orthostatic tolerance in healthy subjects. Moreover, blood pressure and heart rate regulation at rest and during orthostatic stress did not differ between normocaloric and hypercaloric nutrition.

The combination of a carefully controlled dietary intervention and a rigorous methodology for assessing orthostatic tolerance is a strength of our study. We determined orthostatic tolerance through head-up tilt testing combined with lower body negative pressure [10]. This method has been successfully applied in healthy people and in patients with neurally mediated syncope to demonstrate that moderate exercise training [25], sleeping in the head-up position [9], increased salt ingestion [11], and water drinking [30] improve orthostatic tolerance. Based on these studies, we suggest that an improvement in orthostatic tolerance by an average of 5 min could be considered relevant. Our study was powered to show a 2 min difference in orthostatic tolerance between interventions. The dietary intervention was implemented by experienced staff, partly on an outpatient basis, and in the 2 days before orthostatic tolerance testing on a clinical research ward dedicated to highly controlled human investigations [16]. All study participants achieved the increase in caloric intake as planned in the study protocol. Moreover, we controlled important potential confounding variables such as physical activity and salt intake. We are, therefore, confident that our study excludes a relevant change in orthostatic tolerance when modestly increasing energy intake for a few days. The fact that blood pressure and heart rate regulation including baroreflex sensitivity were not affected by the interventions further support this conclusion.

Our findings have to be reconciled with previous investigations showing intense cross talk between metabolic and cardiovascular control through the autonomic nervous system, with effects on orthostatic tolerance. In animal studies, high-fat feeding elicited sympathetic activation [18, 27, 32]. The response is at least partly mediated through the leptin-melanocortin system in the hypothalamic arcuate nucleus [3]. In addition to affecting the cardiovascular system, sympathetic activity raises metabolic rate, thereby maintaining energy balance and body weight [35]. Chronic changes in caloric intake elicit similar responses in susceptible humans. Sympathetic activity is increased in obese individuals compared to lean individuals, even before the onset of obesity-related hypertension [13]. Moreover, experimental dietary weight increases and losses decrease energy expenditure and cardiac sympathetic activity in lean and in obese people [20, 36]. Weight loss through sleeve gastrectomy reduces sympathetic activity [33] and orthostatic intolerance is a common complication following the procedure [5]. Furthermore, hypocaloric nutrition, as in patients with anorexia nervosa, is associated with altered autonomic responses to orthostatic stress consistent with reduced sympathetic activity [36]. Finally, few days of hypocaloric diet decreased orthostatic tolerance in healthy people [12], while a similar surplus of energy intake in our study was ineffective. We speculate that this discrepancy may be the expression of an evolutionary conserved thrifty phenotype, enabling humans to better maintain energy balance and body weight in the face of energy shortages compared with energy excess [26].

An important limitation of our study is that orthostatic tolerance in the laboratory may not always translate to orthostatic symptom load in daily life. Moreover, changes in orthostatic tolerance in healthy people cannot be simply extrapolated to patients with neurally mediated syncope. However, previous studies using a similar approach, such as studies on salt or water intake, elicited similar improvements in orthostatic tolerance in healthy people and in patients with syncope [7, 11, 30]. Moreover, both interventions also prevented presyncope or syncope in real life [1]. Another potential limitation is that the magnitude of the change in caloric intake and the duration of the intervention may not have been sufficient. However, practical reasons and previous investigations led us to test a shorter-term intervention. First, the intent of the study was to identify a possible practical treatment to orthostatic intolerance that could be applied in astronauts before landing on Earth or on another celestial body, as well as in patients with neurally-mediated syncope. A longer duration intervention would be more difficult to implement in this setting. Furthermore, while hypercaloric nutrition leading to weight gain may be sensible in malnourished patients, more prolonged hypercaloric nutrition and weight gain could pose metabolic and cardiovascular risks in people of normal weight. Finally, previous studies have shown that short-term changes in caloric intake can impact cardiovascular function and sympathetic activation. Three days of caloric restriction significantly decreased diastolic function in healthy people [15]. Furthermore, animal studies showed a significant increase of mean arterial pressure after 3 days on a high-fat-diet [4].

Despite these issues, we suggest that modestly increasing caloric intake for a few days is unlikely to improve orthostatic tolerance in normal weight individuals prone to neurally mediated syncope. However, given the intense cross talk between energy balance and cardiovascular autonomic control, influences of more pronounced increases in energy balance on orthostatic tolerance deserve to be studied, particularly in patients who are malnourished and underweight, such as patients with anorexia nervosa or in frail elderly people who are prone to fall-related injuries.
